# Supplementary material for: Infrared absorption spectroscopy of a single polyatomic molecular ion
Source: Nature. 2026 Aug 26;657(8131):364–8. doi: 10.1038/s41586-026-10915-8 (PMC13558049; doi:10.1038/s41586-026-10915-8)
Supplement: Supplementary file 1 [file 41586_2026_10915_MOESM1_ESM.pdf]

nature portfolio

Peer Review File

Infrared absorption spectroscopy of a single polyatomic molecular ion

Corresponding Author: Dr Philipp Schindler

This file contains all reviewer reports in order by version, followed by all author rebuttals in order by version.

Version 0:

Reviewer comments:

Referee #1

(Remarks to the Author)
Report on “Infrared absorption spectroscopy of a single polyatomic molecular ion.”

In recent years, we have seen significant progress in adopting coherent quantum control tools for precision spectroscopy and quantum control of molecules. This growing, exciting, and demanding field of research employs a range of experimental approaches. Using quantum-logic techniques with molecular ions is a promising approach, as it enables the investigation of a variety of naturally occurring molecular ions.

Quantum-logic spectroscopy is a technique in which an atomic ion is used to detect the spectroscopic signature of a molecular ion through their shared motion in the trap. There are different approaches to creating this signature, e.g., sideband pulses, state-dependent forces, energy transfer in inelastic collisions, among others. Here, the authors follow their own proposal (ref. 30 in the paper) to use photon-absorption recoil as their quantum-logic signal.

This approach (detecting the recoil of photon absorption) is novel in the field. It allows the use of ultrashort laser pulses for molecular spectroscopy (other methods, such as sideband pulses or state-dependent forces, use cw lasers). As such, it opens new possibilities for observing rapid molecular dynamics (such as IVR) at the single-molecule level.

To detect the small signal of photon absorption recoil, the authors use a “cat” state interferometer to enhance it. This approach was already demonstrated in proof-of-principle experiments with atomic ions. Here, it is implemented for the first time on molecular ions.

This paper truly advances the field of molecular quantum technologies by demonstrating experimental ultrafast quantum-logic spectroscopy on polyatomic molecular ions. For that, this accomplishment is well-deserving of publication in the Nature journal. However, several issues with the manuscript need to be addressed by the authors before I recommend it for publication.

Main concerns:

1) In Section II, the authors characterize their method using a series of short electric pulses to emulate the absorption events. The authors state (line 196) - “The signal is smaller than what is expected from Equation 4 due to experimental imperfections.” However, Equation 4 is derived after several simplifications of the real experimental situation (not imperfections), which the authors could have included in their theoretical model. For example, the authors should include the out-of-phase mode, avoid the Lamb-Dicke approximation, and include motional-off-resonant terms (see, e.g., Rugoski et. al, Communications Physics 8, 471 (2025), for the role of spectator modes in motional excitation). I wonder if, with these additions, the authors can avoid or justify using an empirical model (Equation 5). Another point to discuss is that the emulation is performed by applying a force on both ions (they are both charged), whereas the photon-absorption kicks are applied only to the molecular ion.

2) In Fig. 2c, the authors compare the “quantum-enhanced” and “direct” methods. However, only for the “quantum-enhanced” process do the authors provide an experimentally measured signal. It would be great to compare both methods experimentally. If I understand correctly, the “direct” method sequence is identical to the “quantum-enhanced” sequence, just

without the “cat”-state creation and annihilation. This addition will also support the claim in lines 124-127.

3) In the main result of this paper, Fig. 5, four experimental points are missing in the absorption spectrum (two red points @ v< 3600 cm^-1 and two red points @ v >= 4000 cm^-1). These points are crucial for understanding the background spectroscopic signal level of the experiment.

4) Throughout the paper, the authors use the phrase “single-photon absorption”. If I understand correctly (see Fig. 4a - saturation of p_abs), the molecule absorbs and stimulates-emit many photons in each sequence (there is no spontaneous emission at this timescale). The recoil of these events cancels out such that the only signature on the crystal momentum is if there was one more absorption than stimulated emission event (only the last photon counts). Referring to this process as “single-photon absorption” may therefore be misleading, and the authors should clarify this distinction explicitly in the text.

5) The authors claim in the abstract, introduction (line 38), and discussion (lines 311, 325) that when the signal is strong enough to facilitate a single-shot measurement, their protocol will represent a quantum non-demolition measurement. I believe this statement is incorrect. A defining feature of QND measurement is that, given the first measurement outcome, a subsequent measurement yields the same result. In the presented scheme, the probability for photon absorption is always 50%, regardless of previous experiments. While the detection of such absorption events can achieve 100% fidelity and allow unambiguous inference of the molecule's state, the measurement outcome itself is not preserved when the protocol is repeated. For that, the protocol doesn’t qualify as QND.

Small remarks:
1) In line 224, “no spontaneous decay occurs during this period” - so the molecular state decay time >> 34/f_rep = 340 us. In line 243, “time between excitation events is long compared to the radiative lifetime of the excited state” - so the molecular state decay time << 10’s ms?. What is the molecular state decay time?

2) Line 181 - “we apply a series of n_kick”. Line 186 - “The displacement magnitude from a single kick pulse is set to”. Fig. 3 caption - “produced by a single electric kick pulse”. Does “single kick pulse” refer to a series of n_kick kicks? or for n_kick=1. Please rephrase for clarity. Also, what is the temporal width of each pulse?

3) In Fig. 3a, there is a small oscillation signal even when no kicks are applied. What is the source of this signal?

4) Error bars - the nature of the error bars in Figs. 3, 4, and 5 are not explained in the figure captions.

Referee #2

(Remarks to the Author)

The authors devise and convincingly demonstrate a novel method of realizing absorption spectroscopy of a single, trapped ion, and demonstrate it on a small triatomic molecular ion, CaOH+. The method is clever and appears to be quite general, exploiting non-classical motional states of the trapped ensemble. The method fits into the increasingly broad suite of powerful ideas that surround Quantum Logic Spectroscopy. In contrast to the [rather limited] earlier experiments that have observed single photon absorption events of single polyatomic molecular ions, this method provides a true projective measurement, as most QLS methods do. The work also provides a clear runway to further exciting work, including true state-prep, and high-precision spectroscopy limited by fundamental physics mechanisms, rather than experimental details.

The method is clear, clever, and convincingly demonstrated, and a nice manipulation of the various “quantum levers” available in this system. It is unambiguous that the authors did what they claimed.

I mildly disagree with the second half of the sentence:
“To date, these experiments are limited to molecular species with relatively simple internal structure and are predominantly applied to diatomic molecules, due to the requirement of high-fidelity molecular state preparation.”
I would say this inverts the requirement and outcome - experiments with diatomic molecular ions - and in particular CaH - YIELD high-fidelity state preparation, rather than require it. In fact this ability is a major motivation for the presented work.

The authors write that their technique is limited to modest alpha by “the actual signal measured at large |α| is limited by atomic and motional decoherence.” I am curious about this, and feel the authors could be more specific. What atomic decoherence are we talking about? The authors imply that the reason large alpha doesn’t work is that the state prep takes longer in this case; this sounds plausible when outracing atomic decoherence (some kind of spontaneous decay? scattering?) but perhaps less plausible for motional decoherence, which likely occurs at a rate which depends directly on alpha. A phenominical model is presented, but the physical origin of this decoherence remains rather unclear. All of which is a long way to say that the origins of the limits on alpha (and thus the sensitivity of the method) are not clear. Is there a path to extend this technique to a SPAM type scenario, where state preparation and readout are done at some modest but reasonable fidelity?

The work was done with an ultrafast, and hence broadband, spectroscopy laser. The authors write that this can be “straightforwardly achieved by implementing our method with narrow-linewidth lasers.” This application, and the associated ultra-high-precision spectroscopy, is a major motivation of this scheme, since it provides a major prerequisite - perhaps the only still needed prerequisite? - for realizing state prep in a polyatomic molecule. As such, I think it could be described in a

bit more detail. In particular, the blessing and curse of polyatomic, and in general complex, molecules is that they occupy a large number of rotational and hyperfine states. How would these states be iterated through in the high resolution experiment? How does this scale with molecule complexity? Is there a path here to performing state prep on highly complex molecules? However, this discussion should be considered optional by the authors; the work they have done stands strongly on its own.

The authors also mention ultrafast pump-probe spectroscopy as an application. This sounds plausible, but I am unclear what advantage the single molecule nature of this work brings to this field.

All told, I find this to be excellent work and recommend it for publication.

Referee #3

(Remarks to the Author)
A. Summary of the key results:
In this manuscript, the authors demonstrate sensitivity to small changes in the motional state of a trapped CaOH+ - Ca+ ion crystal due to recoil of absorbing a photon from excitation of a vibrational transition in the molecule. It is achieved by preparing a coupled motional mode of the ion crystal in a nonclassical cat state, i.e. superposition of motional coherent state, before probing the vibrational transition, and amplifying the signal strength. The sensitivity of this method, originally demonstrated in atomic ions, to minute changes in ion motion is calibrated by applying electrical motional excitation of known strength synchronized to the ion motion. The factors limiting the sensitivity are identified. Using a tunable pulsed laser with the pulses impinging on the molecule in sync with the ions' motion, a coarse excitation spectrum of a vibrational transition is obtained. This work presents the first demonstration of nondestructive single polyatomic ion spectroscopy.

B. Originality and significance:
This work applies a protocol originally proposed in the context of and demonstrated in an atomic ion to vibrational spectroscopy of a polyatomic ion. The preparation of a trapped CaOH+ - Ca+ ion crystal was investigated in a previous publication from the authors. The effect of a vibrational excitation is observed as a small change in motional state due to the recoil from absorbing a single photon. The signal is amplified by employing nonclassical motional states. This work can thus be considered a practical application of quantum-enhanced metrology in molecular physics. It also demonstrates a new approach to study single polyatomic ions nondestructively. The authors listed various speculated impacts of the demonstrated method. However, the narratives that justify them should be made more rigorous.

C. Data & methodology: validity of approach, quality of data, quality of presentation
The authors take a reasonable approach toward realizing the protocol by calibrating the sensitivity using excitation of known temporal behavior and strength. The limitation of the method could thus be identified and quantified. With the polyatomic ion one of the major novelties of the work, it leaves the readers wanting more on results specific to the polyatomic nature of the ion. As it is now, only figure 5 is unique to a polyatomic ion. The rest of the results are from the calibration process. The large width of the feature in Fig. 5 also does not make a strong case of employing the cat state for the motion in the study. The writing of the manuscript is clear and easy to read.

D. Appropriate use of statistics and treatment of uncertainties
No definition for the uncertainties is given. These considerations are not very relevant for this proof-of-principle demonstration.

E. Conclusions: robustness, validity, reliability
This work offers a path to nondestructive spectroscopy of single polyatomic molecular ions. The claim that the resolution can be improved by probing with a narrow linewidth, supposedly continuous-wave, laser appears contradictory to the apparent requirement of ensuring consistent phase between the momentum kick due to photon absorption and the ion motion. The limitation of the method is not explicitly stated. Obvious questions include: to what extent can the excitation probability be significant without molecular state preparation? Does the protocol rely on a broadband light source exciting numerous transitions and covering significant state population in the molecule to ensure acceptable signal-to-noise ratio?

F. Suggested improvements: experiments, data for possible revision
Possible improvements:
1. Elaborate on the prospective of obtaining narrower lines beyond model prediction.
2. Include a discussion on the applicable range of transitions and molecular species.
3. Showing more results specific to the polyatomic nature of the molecule will further strengthen the manuscript.

G. References: appropriate credit to previous work?
The references listed appear adequate.

H. Clarity and context: lucidity of abstract/summary, appropriateness of abstract, introduction and conclusions
The abstract summary does not seem to conform to the Nature format. For example, no references are included. The conclusion and outlook invite some questions, a few of which are listed below.

The referee suggests revising the manuscript before further consideration for publication.

Questions and suggestions:
1. Fig. 1: The zero-point energy for the harmonic potential of the coupled ion motion should be close to half the level spacing.
2. Line 66, the nonclassical states of motion should be for both ions, not just the atom.
3. Fig. 4: It is helpful to include a simple explanation, if possible, on why P_abs does not approach 1.
4. Line 245 and 246: How long is the experimental sequence? What is the time scale for thermalization? Do these numbers affect the number of probe pulses that can be accommodated in a sequence?
5. Line 257: what determines the upper limit of 34 pulses?
6. Line 305 to 307, does high-resolution spectroscopy require state preparation absent in the manuscript? How can the events of photon absorption from a narrow-linewidth laser be synchronized with the ion motion to ensure consistent signal?
7. Line 311: It is not clear how QND measurements are compatible with the demonstrated protocol. The state of a molecule generally changes after absorbing a photon.

Version 1:

Reviewer comments:

Referee #1

(Remarks to the Author)
The authors have addressed all my comments to my satisfaction, and I recommend the paper for publication.
I have a few minor remarks that could improve the readability of the manuscript:
Figure 3 caption – “Error bars are one standard deviation”: it would be helpful to specify the origin of the error bars (e.g., quantum projection noise, fit uncertainty, etc.).
Lines 125–126 – “Due to fundamental and technical noise of the experiment”: please briefly specify the dominant sources of fundamental and technical noise.
Line 130 – “By applying near-resonator”: the word “near” can be omitted, as the excitation is resonant.
Lines 177 and 196 – “Imperfection in the experiment”: please briefly clarify which imperfections are being referred to.

Referee #2

(Remarks to the Author)
The work was strong in the original submission, and is stronger now. In particular, referee #1 made a positive but very detailed review, and the points they made have been adequately and thoroughly addressed. The most important point, in my opinion, was the discussion of QND measurements, which has now been clarified in the methods section.

My (referee #2) critiques have also been adequately addressed. I recommend for publication.

Referee #3

(Remarks to the Author)
In the revision, the authors address the comments of the referees on the first version of the manuscript. Notably, a model incorporating experimentally determined systematic effects is introduced to explain the saturation behavior of the data. An argument and an example protocol for the QND claim are added. The idea of using a narrow-linewidth laser to achieve high-resolution spectroscopy while using the photon recoil as detection signal is explained in more detail. The applicable range of the technique is discussed. The manuscript is thus much improved. However, the response also motivates further discussion。 The authors should address the following comments before the manuscript moves further in the editorial process.
Comments:
1. QND: The QND narrative is not satisfactory. The newly added justification is as if the final state of a measurement is known and, if outside a subspace, can be transferred back to the chosen subspace, such measurement can be deemed QND. This argument seemingly redefines what QND stands for and is not compatible with the convention. The QND claim is not essential for the manuscript and can be omitted or rephrased as nondestructive state detection.
2. Line 267-269: Without state preparation, the initial state before the spectroscopy probe is a mixed state. This mixture seems more likely the reason for the saturation. The laser pulses can possibly have phase coherence within the 350 us molecular excitation time.
3. Methods, eq. 11: the collapse operators appear to be questionable. The time constants should be in square roots, and the collapse operator for motion should not be proportional to the number operator.
4. Methods F: This assumes only two pure quantum states are involved, with rotational and nuclear spin degrees of freedom all resolved. Is the discussion only applicable to situations when a transition between two pure quantum states can be selectively targeted by, say, tailoring the frequency and/or polarization of the probe?

Open Access This Peer Review File is licensed under a Creative Commons Attribution 4.0 International License, which permits use, sharing, adaptation, distribution and reproduction in any medium or format, as long as you give appropriate credit to the original author(s) and the source, provide a link to the Creative Commons license, and indicate if changes were made.
In cases where reviewers are anonymous, credit should be given to 'Anonymous Referee' and the source.
The images or other third party material in this Peer Review File are included in the article's Creative Commons license, unless indicated otherwise in a credit line to the material. If material is not included in the article's Creative Commons license and your intended use is not permitted by statutory regulation or exceeds the permitted use, you will need to obtain permission directly from the copyright holder.
To view a copy of this license, visit https://creativecommons.org/licenses/by/4.0/

We would like to express our sincere appreciation to the referees for their insightful comments and constructive suggestions. We have carefully considered all questions raised by the reviewers, which are hopefully addressed in the following discussions and the revised manuscript. We are also providing the full reviews to provide context for our responses to all referees.

Referee #1 (Remarks to the Author):

Report on “Infrared absorption spectroscopy of a single polyatomic molecular ion.”

In recent years, we have seen significant progress in adopting coherent quantum control tools for precision spectroscopy and quantum control of molecules. This growing, exciting, and demanding field of research employs a range of experimental approaches. Using quantum-logic techniques with molecular ions is a promising approach, as it enables the investigation of a variety of naturally occurring molecular ions.

Quantum-logic spectroscopy is a technique in which an atomic ion is used to detect the spectroscopic signature of a molecular ion through their shared motion in the trap. There are different approaches to creating this signature, e.g., sideband pulses, state-dependent forces, energy transfer in inelastic collisions, among others. Here, the authors follow their own proposal (ref. 30 in the paper) to use photon-absorption recoil as their quantum-logic signal.

This approach (detecting the recoil of photon absorption) is novel in the field. It allows the use of ultrashort laser pulses for molecular spectroscopy (other methods, such as sideband pulses or state-dependent forces, use cw lasers). As such, it opens new possibilities for observing rapid molecular dynamics (such as IVR) at the single-molecule level.

To detect the small signal of photon absorption recoil, the authors use a “cat” state interferometer to enhance it. This approach was already demonstrated in proof-of-principle experiments with atomic ions. Here, it is implemented for the first time on molecular ions.

This paper truly advances the field of molecular quantum technologies by demonstrating experimental ultrafast quantum-logic spectroscopy on polyatomic molecular ions. For that, this accomplishment is well-deserving of publication in the Nature journal. However, several issues with the manuscript need to be addressed by the authors before I recommend it for publication.

We are happy to hear that the referee is of the opinion that our work advances the field and we address and clarify the raised issues below.

Main concerns:

1) In Section II, the authors characterize their method using a series of short electric pulses to emulate the absorption events. The authors state (line 196) - “The signal is smaller than what is

expected from Equation 4 due to experimental imperfections." However, Equation 4 is derived after several simplifications of the real experimental situation (not imperfections), which the authors could have included in their theoretical model. For example, the authors should include the out-of-phase mode, avoid the Lamb-Dicke approximation, and include motional-off-resonant terms (see, e.g., Rugoski et. al, Communications Physics 8, 471 (2025), for the role of spectator modes in motional excitation). I wonder if, with these additions, the authors can avoid or justify using an empirical model (Equation 5). Another point to discuss is that the emulation is performed by applying a force on both ions (they are both charged), whereas the photon-absorption kicks are applied only to the molecular ion.

As the referee pointed out, our noise model in the original manuscript was not motivated with a physical model of the experiment. In the revised manuscript, we compare this heuristic model with a physically motivated one that uses independently measured parameters as input for numerical simulations of the photon absorption technique. We present the results from the simulations in the updated version of Figure 3.(b) and Figure 3.(c) and include a discussion of the experimental imperfections in Methods C.

In our simulation, we analyze the following experimental imperfections: spin dephasing, motional decoherence, atom-light interactions beyond the Lamb Dicke approximation, and spectator modes. We have confirmed that the dominating noise source is motional decoherence, followed by decoherence of the atomic electronic states. By including atomic and motional decoherence, we are able to motivate the empirical prefactor in Equation 5, with atom-light interactions beyond the Lamb Dicke approximation, as shown in Figure 3.(c).

We also estimate the influence of the out-of-phase mode as suggested by the referee. In our case, this effect can be neglected as the out-of-phase mode has been cooled to its ground state before each measurement and affects the operation dominantly via the Debye-Waller coupling. A single photon absorption event acts then as a displacement on the out-of-phase mode with the amplitude of the respective Lamb-Dicke parameter which is similar to the one of the in-phase-mode. The effect of the Debye-Waller coupling on the measurement outcome scales with the square of the mean phonon number which is expected to be below 10^{-4} for our parameters.

With respect to applying the kick to a single or both ions: we are aware that the electric force we introduced in the emulation is applied to both ions while the photon absorption only kicks the molecular ion. However, the photon absorption detection is only sensitive to displacements on the in-phase motional state which are given by the Lamb-Dicke parameters we are using in the manuscript. The electric kick will add different excitation in the out-of-phase mode compared to the photon absorption event, but this can be neglected.

2) In Fig. 2c, the authors compare the "quantum-enhanced" and "direct" methods. However, only for the "quantum-enhanced" process do the authors provide an experimentally measured signal. It would be great to compare both methods experimentally. If I understand correctly, the

“direct” method sequence is identical to the “quantum-enhanced” sequence, just without the “cat”-state creation and annihilation. This addition will also support the claim in lines 124-127.

As the referee suggested, including the experimental measurement for the “direct” method could better showcase the enhancement of the cat-state approach. We attempted to implement the direct method but we could not observe a significant signal-to-noise ratio to make a reliable comparison. The theoretic curve for the “direct” method in figure 3 gives the upper bound for the obtainable experimental signal. To better illustrate this, we replace the theoretic line with a shaded area in the figure to indicate all possible outcomes from the direct measurement.

3) In the main result of this paper, Fig. 5, four experimental points are missing in the absorption spectrum (two red points @ v< 3600 cm^-1 and two red points @ v >= 4000 cm^-1). These points are crucial for understanding the background spectroscopic signal level of the experiment.

We appreciate the referee’s suggestion on this and performed measurements at the corresponding wavelengths as suggested. The results are analyzed and included in an updated version of Figure 5.

4) Throughout the paper, the authors use the phrase “single-photon absorption”. If I understand correctly (see Fig. 4a - saturation of p_abs), the molecule absorbs and stimulates-emit many photons in each sequence (there is no spontaneous emission at this timescale). The recoil of these events cancels out such that the only signature on the crystal momentum is if there was one more absorption than stimulated emission event (only the last photon counts). Referring to this process as “single-photon absorption” may therefore be misleading, and the authors should clarify this distinction explicitly in the text.

The referee is correct that when saturating the transition, one could take the viewpoint that multiple photons will be absorbed and emitted. First, as pointed out by the referee, it is important to notice that the entire pulse train is much shorter than the lifetime of the excited state and these “emission” events are stimulated by the laser rather than spontaneous. The recoil from this stimulated emission is exactly opposite to the one caused by the absorption and the maximum achievable recoil is thus the one of a single absorption event. The referee raises the point that in the saturated regime there are multiple stimulated emission and absorption events. We think that this is an interesting point that is caused by the semiclassical description of the light field.

We want to present an alternative viewpoint: For a fully coherent operation, where the system undergoes Rabi flops instead of saturation, observing these stimulated emission and absorption events would destroy any coherence. We are of the opinion that in this context it would be unusual to talk about multiple emission and absorption events. Overall, we think that this is an interesting discussion but a detailed treatment of this viewpoint would require a full quantum treatment of the light field and would be distracting from our main results and findings. We thus

refrain from adding a detailed discussion in the manuscript. In the interest of being more rigorous, we modified the manuscript and replaced the term “single-photon absorption” with “photon absorption” at multiple instances throughout the manuscript. We also emphasize in the revised manuscript that the method provides sensitivity at the single-photon level rather than that we actually demonstrate single-photon absorption detection.

5) The authors claim in the abstract, introduction (line 38), and discussion (lines 311, 325) that when the signal is strong enough to facilitate a single-shot measurement, their protocol will represent a quantum non-demolition measurement. I believe this statement is incorrect. A defining feature of QND measurement is that, given the first measurement outcome, a subsequent measurement yields the same result. In the presented scheme, the probability for photon absorption is always 50%, regardless of previous experiments. While the detection of such absorption events can achieve 100% fidelity and allow unambiguous inference of the molecule's state, the measurement outcome itself is not preserved when the protocol is repeated. For that, the protocol doesn’t qualify as QND.

We agree with the referee that the technique itself does not implement a QND measurement in a Hilbert space by itself but rather the detection of a transition. However, we want to point out that the recoil detection method can be the basis of a QND measurement which can be implemented in multiple ways and we want to quickly sketch one straightforward possibility here and also in the methods section of the revised manuscript:

We consider a molecular vibrational mode with its three lowest eigenstates |0>, |1>, |2> and want to implement a QND measurement in the |0> - |1> manifold. We assume that we can individually address the |0> - |1> and |1> - |2> transitions with reasonably high fidelity and that the system is initially in a superposition of |0> and |1>. We then transfer population from the |1> to the |2> state while monitoring the photon absorption that can be detected as an atomic excitation (line 163-166), before transferring the population back to |1> again after the detection. Although the detection of a photon absorption event projects the vibration into the |2> state, it is brought down to the state |1> coherently to complete the QND measurement. This scheme is conceptually similar to electron shelving that is used for atomic ions but does not require scattering photons.

We clarified our statement by adding the following sentence:

“With selective single-shot measurement in an effective three-level system, the recoil detection can enable quantum non-demolition measurements on two of the states [34, 35], as discussed in the Methods F.”

in the discussion and added a discussion of the three level QND measurement to the methods section F.

Small remarks:

1) In line 224, “no spontaneous decay occurs during this period” - so the molecular state decay time >> 34/f_rep = 340 us. In line 243, “time between excitation events is long compared to the radiative lifetime of the excited state” - so the molecular state decay time << 10’s ms?. What is the molecular state decay time?

The radiative lifetime of the state is calculated to be around 2.8 ms. We now include the value to justify our claim.

2) Line 181 - “we apply a series of n_kick”. Line 186 - “The displacement magnitude from a single kick pulse is set to”. Fig. 3 caption - “produced by a single electric kick pulse”. Does “single kick pulse” refer to a series of n_kick kicks? or for n_kick=1. Please rephrase for clarity. Also, what is the temporal width of each pulse?

We are referring to the case of n_kick=1 here. We have rephrased the following sentence correspondingly to better clarify this:

“Here, η_k is the displacement magnitude from a single kick pulse which is set to 0.0193(2) to be comparable with the expected recoil of a mid-infrared photon absorption event.”

The width of each electric kick pulse is 10 ns.

3) In Fig. 3a, there is a small oscillation signal even when no kicks are applied. What is the source of this signal?

The amplitude of the oscillation with no kicks is estimated to be 0.014(6) which is comparable to the quantum projection noise of the signal. We thus think it is an artifact from fitting the projection noise to a sinusoidal function. We added a brief explanation in the caption of Figure 3(a) on this:

“...as well as the background signal for n_kick = 0 (orange) due to quantum projection noise.”

4) Error bars - the nature of the error bars in Figs. 3, 4, and 5 are not explained in the figure captions.

The error of the mean excitation shown in Figure 3(a) and Figure 4(c) is quantum projection noise. The uncertainties of the measured signal in Figure 3(b) and Figure 3(c) are from fitting the sinusoidal oscillation amplitude. The error in the estimated effective photon absorption probability shown in Figure 4(a) and Figure 5 is analyzed based on Equation 6. We added brief explanations of the error in Figure 3(a) and Figure 3(b) which should provide the readers sufficient information on the nature of the error bars in the subsequent figures as well. We want to add here that the full analysis code is available online in the linked repository.

Referee #2 (Remarks to the Author):

The authors devise and convincingly demonstrate a novel method of realizing absorption

spectroscopy of a single, trapped ion, and demonstrate it on a small triatomic molecular ion, CaOH+. The method is clever and appears to be quite general, exploiting non-classical motional states of the trapped ensemble. The method fits into the increasingly broad suite of powerful ideas that surround Quantum Logic Spectroscopy. In contrast to the [rather limited] earlier experiments that have observed single photon absorption events of single polyatomic molecular ions, this method provides a true projective measurement, as most QLS methods do. The work also provides a clear runway to further exciting work, including true state-prep, and high-precision spectroscopy limited by fundamental physics mechanisms, rather than experimental details.

The method is clear, clever, and convincingly demonstrated, and a nice manipulation of the various "quantum levers" available in this system. It is unambiguous that the authors did what they claimed.

We are happy to hear that the referee is of the opinion that our results are clever and convincing.

I mildly disagree with the second half of the sentence:
"To date, these experiments are limited to molecular species with relatively simple internal structure and are predominantly applied to diatomic molecules, due to the requirement of high-fidelity molecular state preparation."
I would say this inverts the requirement and outcome - experiments with diatomic molecular ions - and in particular CaH - YIELD high-fidelity state preparation, rather than require it. In fact this ability is a major motivation for the presented work.

We agree with the referee that traditional QLS methods provide a powerful method for state preparation. We removed the statement on the state preparation and the sentence reads now: *"To date, these experiments are limited to molecular species with relatively simple internal structure and are predominantly applied to diatomic molecules"*

The authors write that their technique is limited to modest alpha by "the actual signal measured at large |α| is limited by atomic and motional decoherence." I am curious about this, and feel the authors could be more specific. What atomic decoherence are we talking about? The authors imply that the reason large alpha doesn't work is that the state prep takes longer in this case; this sounds plausible when outracing atomic decoherence (some kind of spontaneous decay? scattering?) but perhaps less plausible for motional decoherence, which likely occurs at a rate which depends directly on alpha. A phenominical model is presented, but the physical origin of this decoherence remains rather unclear. All of which is a long way to say that the origins of the limits on alpha (and thus the sensitivity of the method) are not clear. Is there a path to extend this technique to a SPAM type scenario, where state preparation and readout are done at some modest but reasonable fidelity?

The referee is correct, that a detailed physical error model was missing from our manuscript. We have now performed numerical simulations with independently measured experimental parameters, as discussed in the answer to referee 1 above and in Methods C. The atomic

decoherence being considered here is the dephasing caused by magnetic field fluctuation and the motional decoherence mainly comes from the fluctuation in trap potential due to electric noise. They affect the sensitivity of the measurement not only during the cat state preparation process but also during the 350 μs molecular excitation time.

As the referee pointed out, the method can be implemented in a SPAM scenario for molecular state preparation and readout. Similar to the quantum non-demolition measurement presented in the answer to referee 1 and in Methods F, we can consider a molecular vibrational mode with its three lowest eigenstates |0>, |1>, |2>. By driving the transition between |1> and |2> and monitor the photon recoil, we can projectively prepare the state of the system in |0> based on a negative outcome. Even in the absence of experimental imperfections, the probability of detecting a 2.7 μm photon recoil as an atomic excitation is only around 60% for a cat state amplitude of alpha=15. Therefore, to achieve reasonable fidelity, the process needs to be repeated several times. Our simulations show that achieving a readout fidelity of a molecular transition of around 95% with 10 repetitions requires generating a cat state of alpha=12 with an atomic coherence time of 50 ms and a motional coherence time of 2 s, which is technically challenging but feasible. By replacing the lengthy ground state cooling sequence with a much more time-efficient EIT cooling scheme, we expect that a single repetition under the current cat state generation scheme takes around 1 ms. This can be further reduced to a few hundreds of μs by employing a different, faster cat state creation method, e.g., using multiple ultrafast laser pulses to generate a cat state with spin dependent momentum kicks as demonstrated in [1]. The method can then be used to prepare or detect molecular states with millisecond lifetimes such as the vibrational excitation states.

The work was done with an ultrafast, and hence broadband, spectroscopy laser. The authors write that this can be "straightforwardly achieved by implementing our method with narrow-linewidth lasers." This application, and the associated ultra-high-precision spectroscopy, is a major motivation of this scheme, since it provides a major prerequisite - perhaps the only still needed prerequisite? - for realizing state prep in a polyatomic molecule. As such, I think it could be described in a bit more detail. In particular, the blessing and curse of polyatomic, and in general complex, molecules is that they occupy a large number of rotational and hyperfine states. How would these states be iterated through in the high resolution experiment? How does this scale with molecule complexity? Is there a path here to performing state prep on highly complex molecules? However, this discussion should be considered optional by the authors; the work they have done stands strongly on its own.

The purpose of our statement on narrow-linewidth lasers was a more technical argument: We wanted to state that recoil spectroscopy can be used to measure transitions with a precision that is mostly limited by their linewidth and not by the trap frequency (which has not been surpassed in prior experiments). A narrow linewidth experiment could be implemented by creating a pulse train from a narrow linewidth laser with the repetition frequency being the motional frequency of the ions and a pulse duration that is much shorter than the ion's oscillation period. This enables a coherent momentum kick that can be detected with the cat state and thus allows probing the transition at a linewidth that is limited by the Fourier transform of the pulse train. This interaction

is equivalent to a high-precision interrogation of a transition with a frequency comb which has been demonstrated in atoms and molecules. However, in this regime it is not clear whether recoil spectroscopy can be advantageous over standard quantum logic spectroscopy. An analysis of the optimal method would require further investigation beyond the scope of our manuscript.

We added a more detailed discussion regarding this on page 5:

"A high spectral resolution can be reached by a pulse train originating from a narrow linewidth laser with the repetition period being an integer multiple of the ions' oscillation period and a pulse duration that is shorter than the ion's oscillation period. This enables a coherent momentum kick that can be detected with the cat state while probing the transition at a linewidth that is limited by Fourier transform of the pulse train. This interaction is equivalent to a high-precision interrogation of a transition with a frequency comb which has been demonstrated in atoms and molecules [20]. Naturally, performing high-precision spectroscopy on complex molecules will require state preparation to reach an appreciable signal to noise ratio. We anticipate that recoil detection with spectrally shaped laser sources in combination with traditional quantum logic methods [18] can provide powerful tools to enable such state preparation."

We also agree with the referee that our method on its own is not sufficient to implement high-precision spectroscopy on complex molecules and that it would require additional state preparation. In contrast to broadband time-domain methods, high-precision methods can only be applied to individual (rotational) states. Thus, they require some sort of state preparation to increase the signal to noise ratio. We are of the opinion that a combination of broadband and narrowband techniques will be required to implement this in complex molecules.

We want to give the referee an outlook on some new ideas that might make state preparation in complex polyatomic molecules feasible: We are currently investigating to what extent a vibrational transition can be used to perform laser cooling and optical pumping of the molecular rotation. Compared to electronic transitions, vibrational transitions do not suffer from unfavorable Frank Condon factors. The disadvantage is that cooling and pumping processes will be slow due to the long radiative lifetime which will not be a big issue if the heating rate is much slower. In particular, we are setting up an experiment to use infrared laser pulses with a tailored spectrum to cool and control the rotational degree of freedom of CaOH+, as motivated by [2,3].

The authors also mention ultrafast pump-probe spectroscopy as an application. This sounds plausible, but I am unclear what advantage the single molecule nature of this work brings to this field.

The referee makes a good point: It is indeed less important to apply single-molecule resolution to investigate ultrafast time dynamics than for high-precision experiments as the interaction with the environment might be on longer timescales than the entire pump-probe sequence. The

interaction will thus not add to collisional broadening. However, the combination of using recoil detection and traditional QLS methods for state preparation will enable a more controlled investigation of time dynamics where effects which are washed out in an ensemble averaging can be observed [4].

All told, I find this to be excellent work and recommend it for publication.

Referee #3 (Remarks to the Author):

A. Summary of the key results:
In this manuscript, the authors demonstrate sensitivity to small changes in the motional state of a trapped CaOH+ - Ca+ ion crystal due to recoil of absorbing a photon from excitation of a vibrational transition in the molecule. It is achieved by preparing a coupled motional mode of the ion crystal in a nonclassical cat state, i.e. superposition of motional coherent state, before probing the vibrational transition, and amplifying the signal strength. The sensitivity of this method, originally demonstrated in atomic ions, to minute changes in ion motion is calibrated by applying electrical motional excitation of known strength synchronized to the ion motion. The factors limiting the sensitivity are identified. Using a tunable pulsed laser with the pulses impinging on the molecule in sync with the ions' motion, a coarse excitation spectrum of a vibrational transition is obtained. This work presents the first demonstration of nondestructive single polyatomic ion spectroscopy.

B. Originality and significance:
This work applies a protocol originally proposed in the context of and demonstrated in an atomic ion to vibrational spectroscopy of a polyatomic ion. The preparation of a trapped CaOH+ - Ca+ ion crystal was investigated in a previous publication from the authors. The effect of a vibrational excitation is observed as a small change in motional state due to the recoil from absorbing a single photon. The signal is amplified by employing nonclassical motional states. This work can thus be considered a practical application of quantum-enhanced metrology in molecular physics. It also demonstrates a new approach to study single polyatomic ions nondestructively. The authors listed various speculated impacts of the demonstrated method. However, the narratives that justify them should be made more rigorous.

We believe that our narratives are more rigorous in the revised manuscript.

C. Data & methodology: validity of approach, quality of data, quality of presentation
The authors take a reasonable approach toward realizing the protocol by calibrating the sensitivity using excitation of known temporal behavior and strength. The limitation of the method could thus be identified and quantified. With the polyatomic ion one of the major novelties of the work, it leaves the readers wanting more on results specific to the polyatomic nature of the ion. As it is now, only figure 5 is unique to a polyatomic ion. The rest of the results are from the calibration process. The large width of the feature in Fig. 5 also does not make a strong case of employing the cat state for the motion in the study. The writing of the manuscript is clear and easy to read.

As the referee points out below, the manuscript describes a proof-of-principle implementation of a method that can be readily applied to a wide variety of polyatomic molecules. We think that the novelty here lies in the method itself and not in the investigation of a specific complex polyatomic molecule.

D. Appropriate use of statistics and treatment of uncertainties

No definition for the uncertainties is given. These considerations are not very relevant for this proof-of-principle demonstration.

We added descriptions of the uncertainties in the figure captions. We want to add here that the entire data analysis code is available in the linked online repository.

E. Conclusions: robustness, validity, reliability

This work offers a path to nondestructive spectroscopy of single polyatomic molecular ions. The claim that the resolution can be improved by probing with a narrow linewidth, supposedly continuous-wave, laser appears contradictory to the apparent requirement of ensuring consistent phase between the momentum kick due to photon absorption and the ion motion. The limitation of the method is not explicitly stated. Obvious questions include: to what extent can the excitation probability be significant without molecular state preparation? Does the protocol rely on a broadband light source exciting numerous transitions and covering significant state population in the molecule to ensure acceptable signal-to-noise ratio?

We agree that using a narrow linewidth laser to realize coherent momentum kicks seems counter intuitive. However, one can apply multiple pulses originating from a narrow linewidth laser to implement coherent kicks while the Fourier limit of the pulse train is much smaller than the ion's motional frequency. This works, if the pulses are separated by multiples of the ions' oscillation period and if a single pulse is much shorter than the oscillation period. In our case, it would be sufficient to implement pulses with a duration as long as 100ns which can be realized with usual acousto-optical modulators. We added a description of this to the manuscript on page 5:

"A high spectral resolution can be reached by a pulse train originating from a narrow linewidth laser with the repetition period being an integer multiple of the ions' oscillation period and a pulse duration that is shorter than the ion's oscillation period. This enables a coherent momentum kick that can be detected with the cat state while probing the transition at a linewidth that is limited by Fourier transform of the pulse train. This interaction is equivalent to a high-precision interrogation of a transition with a frequency comb which has been demonstrated in atoms and molecules [20]. Naturally, performing high-precision spectroscopy on complex molecules will require state preparation to reach an appreciable signal to noise ratio. We anticipate that recoil detection with spectrally shaped laser sources in combination with traditional quantum logic methods [18] can provide powerful tools to enable such state preparation."

We have clarified some of the other limitations of our method already in more detail in the answers of referee 1 and 2. With respect to the specific questions of the referee: We expect that

for a broadband laser pulse without state preparation, the excitation probability can approach 100% with sufficient laser pulse energy to excite the molecule with a single laser pulse, and with molecular alignment techniques to avoid variation in excitation efficiency due to random orientation of the molecule [5]. For high-precision experiments, a more complete state preparation will be required as discussed in the answers to referee 2.

F. Suggested improvements: experiments, data for possible revision
Possible improvements:
1. Elaborate on the prospective of obtaining narrower lines beyond model prediction.

The precision of our theoretical prediction is expected to be around 10 cm^-1. This value largely arises from an approximation used to describe the coupling of the O-H stretching mode with the Ca-O-H bending and O-H stretching vibrational modes. It can be readily reduced by rigorously solving the vibrational problem, leaving uncertainties associated with orbital basis-set and coupled-cluster expansion incompleteness of around 1.5 and 2 cm^-1, respectively. Further reduction to below 1 cm^-1, although possible, will require extensive work beyond the current state of the art in electronic-structure theory, as well as the inclusion of relativistic, nonadiabatic, and QED effects. By applying the high-resolution implementation described in our previous answers to referee 2 and 3, however, we should be able to achieve a linewidth that is limited by the laser linewidth and can be much lower than 1 cm^-1. We thus believe that such high-precision experiments may trigger progress in the theory of polyatomic molecules.

2. Include a discussion on the applicable range of transitions and molecular species.

We thank the referee for this suggestion. The range of applicable transitions is determined by the magnitude of the momentum kick when absorbing a photon and thus the wavelength of the transition. The magnitude of the kick is represented by the Lamb Dicke parameter which scales as the inverse square root of the transition frequency. We think that with our parameters, it will be difficult to target transition frequencies below 2000 cm-1. Measuring vibrational transitions beyond this would require experimental efforts on increasing the magnitude of the kick by lowering the trap frequency or increasing the amplification of the recoil signal. In terms of molecular species, we are working with calcium ions of 40 amu, which limits the mass of the molecular species that we can reliably confine and work with to below 100 amu. Furthermore, the radiative lifetime should ideally be longer than the time required to create the cat state in order to achieve maximum detection efficiency.

We added a paragraph at the end of the main text on this:

“The presented method can be applied to any molecular transition that produces a momentum kick of sufficient magnitude based on Equation 1. With our set of parameters, the method should be able to detect photon absorption events on transitions with a frequency down to 2000 cm^-1. Probing transition frequencies beyond this requires better signal-to-noise ratio which is possible with, e.g., better atomic and motional coherence. The transitions considered in the discussion here are limited to vibrational transitions where the radiative lifetime of the excited states are much longer than time to create the cat state. If this is not the case, the momentum kick has

also a diffusive part that reduces the achievable detection efficiency [21], which applies to dipole allowed transitions in the visible and UV part of the spectrum."

3. Showing more results specific to the polyatomic nature of the molecule will further strengthen the manuscript.

We agree with the referee that investigating hallmark features of polyatomics is one of the main motivations for our method. A clear signature of polyatomic behavior would be the demonstration of intramolecular redistribution among multiple vibrational modes. We plan to implement this using time domain pump-probe techniques as proposed in [6]. However, we currently lack the laser power to implement single probe and pump pulses that provide enough excitation of the vibrational modes. We are in the process of upgrading our laser system to enable these measurements but we think that demonstrating this is beyond the scope of the present manuscript.

G. References: appropriate credit to previous work?
The references listed appear adequate.

H. Clarity and context: lucidity of abstract/summary, appropriateness of abstract, introduction and conclusions
The abstract summary does not seem to conform to the Nature format. For example, no references are included. The conclusion and outlook invite some questions, a few of which are listed below.

The referee suggests revising the manuscript before further consideration for publication.

Questions and suggestions:
1. Fig. 1: The zero-point energy for the harmonic potential of the coupled ion motion should be close to half the level spacing.

We modified Figure 1 accordingly.

2. Line 66, the nonclassical states of motion should be for both ions, not just the atom.

We modified the sentence as suggested:

"However, it is possible to amplify the signal of such a recoil by exploiting non-classical states of motion of both ions. "

3. Fig. 4: It is helpful to include a simple explanation, if possible, on why P_abs does not approach 1.

We added a sentence to explain this briefly (line 270-273) : *"The absorption probability is expected to saturate to 0.5 as the number of laser pulses increases based on the model due to the phase incoherence between the laser pulses."*

4. Line 245 and 246: How long is the experimental sequence? What is the time scale for thermalization? Do these numbers affect the number of probe pulses that can be accommodated in a sequence?

As illustrated in Figure 4.(b), the experimental sequence contains: atomic and motional state preparation (about 25ms, mostly ground state cooling of the crystal), cat state generation pulse (175 μs), molecular excitation time (350 μs for 34 femtosecond laser pulses), cat state reversal pulse (175 μs), atomic σx(π/2) pulse (7 μs), atomic state detection (500 μs). The radiative lifetime of the first excitation state of the molecular vibration is around 3ms, which is much longer than the current molecular excitation time and cat state reversal pulse duration combined. This assures that the effect of the photon recoil due to the spontaneous emission can be neglected with current experiment sequence. Considering the repetition rate of our femtosecond laser (100 kHz), the actual limit on the number of femtosecond laser pulses that can be accommodated in the sequence should be around 100.

5. Line 257: what determines the upper limit of 34 pulses?

The limit of 34 pulses was chosen arbitrarily. We did not try higher numbers given that the signal was already saturated.

6. Line 305 to 307, does high-resolution spectroscopy require state preparation absent in the manuscript? How can the events of photon absorption from a narrow-linewidth laser be synchronized with the ion motion to ensure consistent signal?

We added discussion on these points on page 5:

"A high spectral resolution can be reached by a pulse train originating from a narrow linewidth laser with the repetition period being an integer multiple of the ions' oscillation period and a pulse duration that is shorter than the ion's oscillation period. This enables a coherent momentum kick that can be detected with the cat state while probing the transition at a linewidth that is limited by Fourier transform of the pulse train. This interaction is equivalent to a high-precision interrogation of a transition with a frequency comb which has been demonstrated in atoms and molecules [20]. Naturally, performing high-precision spectroscopy on complex molecules will require state preparation to reach an appreciable signal to noise ratio. We anticipate that recoil detection with spectrally shaped laser sources in combination with traditional quantum logic methods [18] can provide powerful tools to enable such state preparation."

7. Line 311: It is not clear how QND measurements are compatible with the demonstrated protocol. The state of a molecule generally changes after absorbing a photon.

We added a discussion on how to implement an exemplary QND measurement in the manuscript and the methods as already outlined in the replies to the first two referees.

[1] K. G. Johnson et al., Nat. Comm. 8, 697 (2017)

[2] P. F. Staanum et al., Nature Physics 6, 271–274 (2010)

[3] P. R. Stollenwerk et al., Phys. Rev. Lett. 125, 113201 (2020)

[4] M. Maiuri, J. Am. Chem. Soc. 142, 1, 3–15 (2020)

[5] H. Stapelfeldt and T. Seideman, Rev. Mod. Phys. 75, 543

[6] P. Schindler, New J. Phys. 21, 083025 (2019)

Dear Editorial Board,

We are delighted to hear that the manuscript can, in principle, be accepted. We have addressed the comments of the Referees and made corresponding changes to the manuscript. As suggested by the editor, we also modified the last sentence of the abstract to read: *"This method* ***can provide a way of performing non-destructive state detection for the measurement and preparation of the quantum state of a wide range of molecular species."*** In addition, we combined Figure 4 and Figure 5 to improve the use of space. We hope that the revised manuscript is now suitable for publication.

Regarding the discussion on quantum non-demolition (QND) measurements, we would like to retain the discussion on non-destructive measurements based on our method as it will be essential for future applications of the method. While we do not strictly agree with Referee #3 that the measurement we proposed in Methods F is not a QND measurement in the 0-1 subspace, we acknowledge that the usage of the term "QND" might be confusing. We have therefore changed the wording to "non-destructive measurement" throughout the manuscript.

Our responses to the remaining issues raised by the Referees are as follows:

Referees' comments:

Referee #1 (Remarks to the Author):

The authors have addressed all my comments to my satisfaction, and I recommend the paper for publication.
I have a few minor remarks that could improve the readability of the manuscript:

Figure 3 caption – "Error bars are one standard deviation": it would be helpful to specify the origin of the error bars (e.g., quantum projection noise, fit uncertainty, etc.).
We modified the description here to:
"Error bars are one standard deviation ***due to quantum projection noise."***
"Error bars are one standard deviation ***from the least-squares fit covariance."***

Lines 125–126 – "Due to fundamental and technical noise of the experiment": please briefly specify the dominant sources of fundamental and technical noise.
We modified the sentence to:
"In practice, this signal is too small to be measured with confidence due to fundamental and technical noise of the experiment ***such as quantum projection noise and imperfect motional ground state cooling."***

Line 130 – "By applying near-resonator": the word "near" can be omitted, as the excitation is resonant.
The term "near-resonant" here refers to the fact that the bichromatic light field is detuned from the atomic qubit transition by exactly the motional frequency of the ions. This is indeed

confusing given the fact that the light field is actually on resonance with respect to the motional sideband of the atomic transition and introduces resonant motional excitation. We decided to remove it as the description here should be clear without specifying the resonance condition.

Lines 177 and 196 – "Imperfection in the experiment": please briefly clarify which imperfections are being referred to.
We made the following adjustment at Line 178-180:
"In order to analyze the effect of imperfections in the experiment on this method ***(mainly due to atomic and motional decoherence)****, we emulate the absorption event by a displacement caused by an electric force on the ion crystal."*
We think this also helps clarify the origin of the imperfections mentioned in the second sentence.

Referee #2 (Remarks to the Author):

The work was strong in the original submission, and is stronger now. In particular, referee #1 made a positive but very detailed review, and the points they made have been adequately and thoroughly addressed. The most important point, in my opinion, was the discussion of QND measurements, which has now been clarified in the methods section.

My (referee #2) critiques have also been adequately addressed. I recommend for publication.

Referee #3 (Remarks to the Author):

In the revision, the authors address the comments of the referees on the first version of the manuscript. Notably, a model incorporating experimentally determined systematic effects is introduced to explain the saturation behavior of the data. An argument and an example protocol for the QND claim are added. The idea of using a narrow-linewidth laser to achieve high-resolution spectroscopy while using the photon recoil as detection signal is explained in more detail. The applicable range of the technique is discussed. The manuscript is thus much improved. However, the response also motivates further discussion。 The authors should address the following comments before the manuscript moves further in the editorial process.
Comments:
1. QND: The QND narrative is not satisfactory. The newly added justification is as if the final state of a measurement is known and, if outside a subspace, can be transferred back to the chosen subspace, such measurement can be deemed QND. This argument seemingly redefines what QND stands for and is not compatible with the convention. The QND claim is not essential for the manuscript and can be omitted or rephrased as nondestructive state detection.
We removed the term "quantum non-demolition" from the text and replaced it with *"nondestructive state detection"*.

2. Line 267-269: Without state preparation, the initial state before the spectroscopy probe is a mixed state. This mixture seems more likely the reason for the saturation. The laser pulses can possibly have phase coherence within the 350 us molecular excitation time.
The referee is correct that the initial state of the molecule before the measurement, while being in the vibrational ground state, is a mixed state of rotation. This is considered in our simulation by randomizing the molecular orientation before applying each laser pulse, which modifies the light-molecule interaction strength accordingly as shown in Equation 14. Furthermore, a mixed rotational state will add additional dephasing due to J-dependent transition frequencies. On top of this, we also expect that the phase of the laser pulses jitters from pulse to pulse due to the lack of repetition rate stabilization.

We modified our statement here to: *"This allows us to compare the experimental data to a simple theoretical model with no free parameters,* ***which predicts the absorption probability to saturate to 0.5 due to the random orientation and dephasing (see Methods E)****"*, and included a brief discussion in Methods E:
"*We also assign the phase ϕ of the light field with respect to the molecular transition randomly, considering the lack of phase stability of the laser from pulse to pulse and the dephasing due to the molecule's mixed rotational state.*"

3. Methods, eq. 11: the collapse operators appear to be questionable. The time constants should be in square roots, and the collapse operator for motion should not be proportional to the number operator.
We appreciate the referee for pointing out this typo and the time constants should indeed be in square roots. However, we think the collapse operator for motion should actually be proportional to the number operator as it originates from the coupling of the system to a phase reservoir. This is used to simulate motional frequency fluctuations due to noise on the static confining electric field. The collapse operator that describes motional heating is not included here, as the heating rate is measured to be around 1 quanta/s in our setup and motional heating can thus be ignored at the time scale of the measurement.

4. Methods F: This assumes only two pure quantum states are involved, with rotational and nuclear spin degrees of freedom all resolved. Is the discussion only applicable to situations when a transition between two pure quantum states can be selectively targeted by, say, tailoring the frequency and/or polarization of the probe?
The analysis in the methods assumes that it is possible to implement high fidelity operations within the respective vibrational subspaces with a single laser pulse. This does not necessarily imply that the molecule is prepared in a single pure state. Especially if the laser pulse is still in the femto- to picosecond regime, optimal control can enable such operations even when the initial state is a mixture of multiple rotational states.
